# Harmonic Extraction in Graphene: Monte Carlo Analysis of the Substrate Influence

**DOI:** 10.3390/ma14175108

**Published:** 2021-09-06

**Authors:** Elena Pascual, José M. Iglesias, María J. Martín, Raúl Rengel

**Affiliations:** Department of Applied Physics, University of Salamanca, E-37008 Salamanca, Spain; josem88@usal.es (J.M.I.); mjmm@usal.es (M.J.M.); raulr@usal.es (R.R.)

**Keywords:** Monte Carlo, graphene, substrate, SiO_2_, h-BN, Al_2_O_3_, high-order harmonics, noise, fluctuations, terahertz

## Abstract

Graphene on different substrates, such as SiO${}_{2}$, h-BN and Al${}_{2}$O${}_{3}$, has been subjected to oscillatory electric fields to analyse the response of the carriers in order to explore the generation of terahertz radiation by means of high-order harmonic extraction. The properties of the ensemble Monte Carlo simulator employed for such study have allowed us to evaluate the high-order harmonic intensity and the spectral density of velocity fluctuations under different amplitudes of the periodic electric field, proving that strong field conditions are preferable for the established goal. Furthermore, by comparison of both harmonic intensity and noise level, the threshold bandwidth for harmonic extraction has been determined. The results have shown that graphene on h-BN presents the best featuring of the cases under analysis and that in comparison to III–V semiconductors, it is a very good option for high-order harmonic extraction under AC electric fields with large amplitudes.

## 1. Introduction

High-order harmonic generation has been proved as an effective way of reaching the THz range not only for traditional semiconductors [1,2,3] but also for two-dimensional materials such as graphene [4,5]. After its public recognition [6], a deep exploration of graphene capabilities was carried out. On the other hand, the relevance of THz regime applications in the last decades has been boosted [7,8,9,10]. In order to achieve high-order harmonic generation in the most effective way, intrinsic high-frequency noise must be analysed too, since it can certainly limit the practical utility of a given material by masking the emerging harmonics [11]. The individual carrier instantaneous velocity at a given time is related to the regular response of the carriers to the field conditions. Furthermore, it must be taken into account that the instantaneous ensemble-averaged velocity is formed by the regular contribution, which consists of odd harmonics of the fundamental frequency due to the collective carrier response, and the fluctuating noise components in the whole frequency range. In an attempt of understanding the physical mechanisms related, it is a priority to examine both contributions [12]. The efficiency of graphene for high-order harmonic generation has already been revealed [4], and tunable resonances characterised by large Q-factors have been observed in the THz regime [13]; on the other hand, we have previously evidenced the potentiality of free-standing graphene, concluding that it is possible to reach the THz range in alike conditions than III–V materials [5]; however, attending to the actual requirements of the experimental works, it is important to develop these analyses for monolayer graphene on different substrates. Ensemble Monte Carlo (MC) simulations [12] have been used for this aim, such modelling tools being highly convenient for determining and studying parameters such as velocity correlation function or noise temperature [14]. The great advantage of the MC procedure is that the sources of fluctuations are intrinsically assimilated through the stochastic nature of the different scattering events. Furthermore, MC simulation has allowed determining the cut-off frequency of the negative differential mobility of graphene over several substrates, observing that they belong to the THz regime for graphene on hexagonal boron nitride (h-BN), silicon carbide (SiC), silicon dioxide (SiO${}_{2}$), and even on hafnium dioxide (HfO${}_{2}$) with similar values to those obtained for III–V nitrides [15].

**Figure 1:**
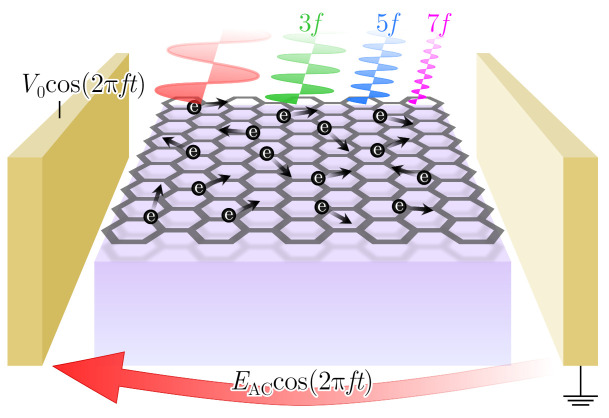
Depiction of the emission of harmonic radiation as a consequence of the oscillating electric field applied over a monolayer graphene sample.

In the present paper, the instantaneous drift velocity fluctuations of graphene on several substrates as a response to the oscillating electric field applied have been inspected; a suspended graphene sample has also been simulated for the sake of comparison. The electric field obeys the following dependence with time:

(1) $$E\left(t\right)=E_{AC}\cos\left(2\pi ft\right)$$

with $E_{AC}$ being the amplitude, and *f* the excitation frequency. As a consequence of the non-linear response of the carriers to this field, the ensemble-averaged drift velocity contains the harmonics of the fundamental signal, and as portrayed in Figure 1, the material may emit radiation at these frequencies. Under these high-order harmonic generation conditions, we will also be able to examine the intrinsic noise by means of our ensemble MC simulator.

The outline of the paper is the following: In Section 2, the main details of the MC simulator employed to extract high-order harmonics are described. In Section 3, the results of the MC simulations of graphene on different substrates are discussed in order to evaluate its efficiency at the THz range. Finally, the main conclusions are drawn in Section 4.

## 2. Materials and Methods

The Monte Carlo simulations have been carried out considering an ensemble of carriers ($10^{4}$ super-particles and an extrinsic concentration of $10^{12}$ cm${}^{-2}$) under the action of an alternating electric field and the consideration of the main scattering mechanisms: Intrinsic optical and acoustic phonons are considered by means of the first-order deformation potential approximation, with the proper parameter fitting so the first-principles calculations in the density functional theory are matched [16]. Carrier–carrier interactions are included by a screened Coulomb potential, applying the Lindhard polarizability in the static limit to calculate the screening function [17,18]. The effect of out-of-equilibrium phonon population, also known as hot phonons, has been implemented in the model, taking into account the number of phonon emissions and absorptions resulting from its scattering with electrons in a gridded momentum space [12]. Finally, surface polar phonons (SPP) scattering has been treated by suitably recording the angle dependence of the scattering probability integrand, considering the Frölich nature of this interaction. Thus, the complete expression for the SPP scattering probability for monolayer graphene considered is the following [19,20]:

(2) $$\Gamma_{SPP,\nu }\left(\varepsilon \right)=\frac{e^{2}\omega_{\nu }\beta }{8\pi \epsilon_{0}{\left(\hbar \nu_{F}\right)}^{2}}|\varepsilon \mp \hbar \omega_{\nu }|\left[n_{\nu }+\frac{1}{2}\pm \frac{1}{2}\right]\sum_{s^{\prime }}\int_{0}^{2\pi }\frac{\exp\left(-2|q|d_{VdW}\right)}{q\epsilon \left(|q|,\omega \right)}F\left(s,k,s^{\prime },k^{\prime }\right)d\theta_{k^{\prime }}$$

being ε the carrier energy, ϵ the dielectric function, ν identifies the phonon mode, *k* the carrier wavevector, $q=k-k^{\prime }$ the exchange vector, *s* the sub-band, $F\left(s,k,s^{\prime },k^{\prime }\right)$ the wavefunction overlap [21], and $v_{F}$ the Fermi velocity. $\mp \hbar \omega_{\nu }$ is the energy shift of the electron due to the absorption (+) or emission (−) of a phonon with the corresponding vibrational frequency at such wavevector, $\omega_{\nu }\left(q\right)$. $n_{\nu }$ is the phonon occupancy at *q*, and the factors $n_{\nu }\left(q\right)$ and $n_{\nu }\left(q\right)+1$ are the annihilation and creation operators related to absorption and emission processes, respectively. $d\theta_{k^{\prime }}$ is the angle between 0 and 2π for each energy value. $d_{VdW}$ is the separation between the graphene layer and the substrate, the Van der Waals distance, typically considered as 0.4 nm in monolayer graphene. Finally, β is a constant defined in the Frölich coupling constant, $F_{\nu }$, related to $\kappa_{0}^{↓}$ and $\kappa_{0}^{↑}$ the low-frequency, and $\kappa_{\infty }^{↓}$ and $\kappa_{\infty }^{↑}$ the high-frequency dielectric constants of the bottom and top substrates. ω is the normalised area of graphene [22]:

(3) $$F_{\nu }^{2}=\frac{\hbar \omega_{\nu }}{2\Omega \epsilon_{0}}\left[\frac{1}{\kappa_{\infty }^{↓}+\kappa_{\infty }^{↑}}-\frac{1}{\kappa_{0}^{↓}+\kappa_{0}^{↑}}\right]=\frac{\hbar \omega_{\nu }}{2\Omega \epsilon_{0}}\beta$$

Table 1 gathers the values considered for the phonon energies and the frequency dielectric constants for the different substrates.

It must be specified that the possible impurities or defects related to the presence of the underlying substrate have been neglected. A deeper description of the simulator is available in [5,12,24].

The timestep considered in the present work is 2 fs or less depending on the simulation conditions. Room temperature and homogeneous local conditions for temperature, carrier concentration and applied electric field have been established, following a material simulation scheme. The Pauli exclusion principle is treated by means of a rejection technique [25]. On the other hand, the hot phonon population coupling accounting for phonon emission and absorption is considered as a result of electron-phonon scattering and phenomenological decay with characteristic times in the relaxation time approximation [12,26].

In the present work, we will mainly focus on the analysis of the intrinsic noise of this material supported on the different substrates under high-order harmonic generation conditions by means of our MC simulator. Furthermore, due to the non-stationary processes induced by the oscillatory field, the study of noise associated with carrier velocity fluctuations requires the use of a two-time correlation function [11]. Therefore, the spectral behaviour of both average and noise contributions of the velocity can be studied to examine the high-order harmonic extraction potentiality and their respective threshold bandwidth. A thorough description of this procedure can be found in [5].

## 3. Results

**Figure 2:**
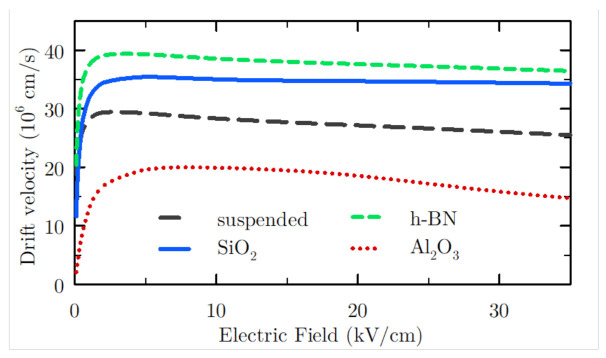
Drift velocity versus applied electric field curves for samples of suspended graphene and graphene on different substrates for T=300 K.

The first step in the search for the best conditions for the generation of high-order harmonics is the study of the velocity-electric field curves of the different samples analysed. A non-linear dependence between both magnitudes is preferable in order to favour the harmonic generation. As it can be seen in Figure 2 for graphene over three different substrates and for the suspended case, a drastic transition occurs from a region with linear velocity-field response to a saturation (and even negative differential resistance) region. The drift velocity tends to saturate for electric fields of about 1 kV/cm, while in the case of Al${}_{2}$O${}_{3}$, the transition to this regime is slightly softer than in the other cases. We note that, in principle, and assuming that the ensemble carrier velocity responds instantaneously to the electric field, a sharper transition between both regions would lead to higher harmonic generation. However, as we will see later, more effects must be taken into account in this analysis. It can also be highlighted that the presence of SPP interactions significantly affects the drift velocity values as well as their dependence on the electric field. This implies that other magnitudes are also affected due to their correlation to the drift velocity, such as the low-field mobility, the velocity saturation and the negative differential conductance at high electric fields. It has been proved that the presence of the substrate drastically reduces the elevated intrinsic low-field mobility; however, the saturation velocity of graphene at high fields is enhanced with certain substrates, such as h-BN and SiO${}_{2}$, as is seen in Figure 2. The strongly anisotropic nature of SPP scattering is the origin of this behaviour, as it was previously discussed in [27,28]. In those works, it was shown that the inelasticity and anisotropy of this phenomenon induces a narrower momentum distribution function with less significant negative tail and average carrier energy, thus yielding enhanced velocity values at high fields in supported samples, such as h-BN and SiO${}_{2}$. However, in the case of Al${}_{2}$O${}_{3}$, we observe that the drift velocity presents lower values than in the suspended sample; in this case, the great relevance of SPP scattering mechanisms as compared to the other substrates implies that deeper considerations should be made in order to understand this behaviour, as we will discuss later.

**Figure 3:**
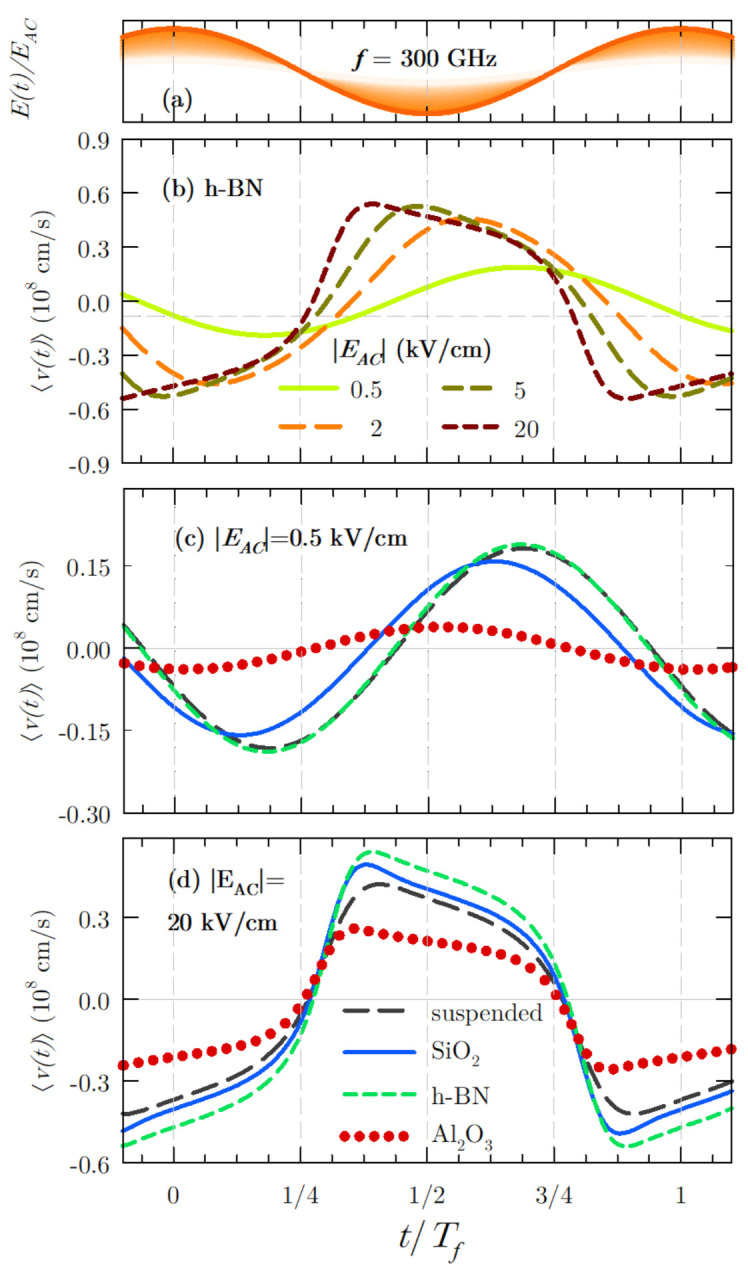
(**a**) Reference of the applied electric field. (**b**) Global velocity response of the system, 〈v(t)〉 for fields of 0.5,2,5 and 20 kV/cm for graphene over h-BN. (**c**) Comparison of the response for the graphene samples under study (on three substrates and suspended) with $E_{AC}=0.5$ kV/cm and (**d**) with $E_{AC}=20$ kV/cm. The excitation frequency is f=300 GHz.

The drift velocity time dependence at a fixed frequency (f=300 GHz) is shown in Figure 3: under different applied electric fields in the case of graphene over h-BN (in (b), as we have previously seen, it is the substrate that yields the largest drift velocity), and also for two fixed fields (0.5 and 20 kV/cm, depicted in Figure 3c,d respectively) but on several substrates, including also suspended graphene for comparison. As it can be observed for the h-BN substrate (see Figure 3b), there is a non-linear response of the drift velocity with the applied electric field. This is due to the electric field-drift velocity relation (non-linear even for low electric field values, as previously seen) and the relaxation characteristics of this material [5]. At the frequency value under study, the velocity response is sinusoidal-like only for very weak electric field amplitude, showing a strong delay in comparison to the oscillatory electric field (shown in Figure 3a). This dephasing between the driving electric field and velocity response is a consequence of carrier inertia. The inertia is lost (i.e., the carrier velocity response adapts more quickly to the field conditions) as the distribution function is hotter due to the application of stronger fields, leading to an increased scattering activity and faster carrier momentum and energy relaxation.

Let us consider the rest of the cases under study. The dephasing at weak field amplitude conditions is also observed for the suspended sample and for graphene on SiO${}_{2}$, although in this latter case, it is slightly reduced (Figure 3c). On the other hand, the velocity response of the system for graphene on Al${}_{2}$O${}_{3}$ presents almost no dephasing with the oscillatory field.

In order to understand these differences, we evaluate the information provided by the MC simulations about the average number of scattering events suffered by the carriers. In Table 2, we show the information for the most relevant scatterings mechanisms (carrier–carrier interaction and SPP) at a small AC electric field amplitude (0.5 kV/cm). For graphene on h-BN and on SiO${}_{2}$, the largest contribution comes from the carrier–carrier interaction, followed by the SPP scattering mechanisms. The SiO${}_{2}$ substrate presents more SPP scattering activity than h-BN. Suspended graphene presents the largest amount of electron–electron interaction. Meanwhile, for graphene on Al${}_{2}$O${}_{3}$, the greatest contribution to scattering mechanisms by far is related to SPP. It must be highlighted that SPP scattering has a strong relaxation ability, yielding a decrease in the carrier energy and, at the same time, not allowing direct backscattering. On the other hand, carrier–carrier interaction changes the velocity and the wavevector orientation of the carriers in the same direction of the applied field but keeps their total energy and wavevector; therefore, at low fields, where the clouds of electrons are very cramped, there is a scarce possibility of energetic exchange. This means that electron–electron interactions do not present a great influence at weak field conditions. On the contrary, the effect of SPP scatterings is meaningful, explaining the absence of dephasing for graphene on Al${}_{2}$O${}_{3}$, where the number of SPP scattering suffered is really high, and the reduced dephasing for graphene on SiO${}_{2}$ substrate as compared to the rest of the cases.

When the value of the electric field amplitude $E_{AC}$ increases, the carrier response is faster, also turning into a more evident square-like shape. This behaviour can be observed not only for graphene on h-BN (Figure 3b) but also for the other cases under study (Figure 3d). At high field conditions, the largest velocity values reached by the system are obtained for graphene on h-BN, which is coherent with the comparison shown in Figure 2. Furthermore, a maximum related to velocity overshoot appears for the different cases under study. Such effect is more noticeable at lower frequencies, as already observed for free-standing graphene [5].

If we consider the number of the main scattering events suffered at high AC electric field (20 kV/cm, presented in Table 3), again, the most important contribution comes from carrier–carrier interaction and SPP. For graphene on h-BN, we observe that both contributions are similar, with a larger presence of SPP scatterings. For graphene on SiO${}_{2}$, similar values of carrier–carrier interaction mechanisms than in the previous case appear, while greater SPP scattering amounts emerge at high field amplitude. The suspended sample presents the largest amount of electron–electron interaction all over the studied field range in comparison to the other cases, while graphene on Al${}_{2}$O${}_{3}$ has the lowest. On the other hand, Al${}_{2}$O${}_{3}$ keeps on with its preponderance of SPP scatterings.

While at the low field, the influence of carrier–carrier interaction was not very important, and at the high field, it is more relevant because the energetic exchange may be larger due to the existence of empty available states in the reciprocal space, thus yielding a change of velocity of the colliding electron pairs. Moreover, although backscattering does not takes place in SPP scatterings, they may foster significant reorientations of the carriers wavevectors as there is a large amount of these mechanisms.

**Figure 4:**
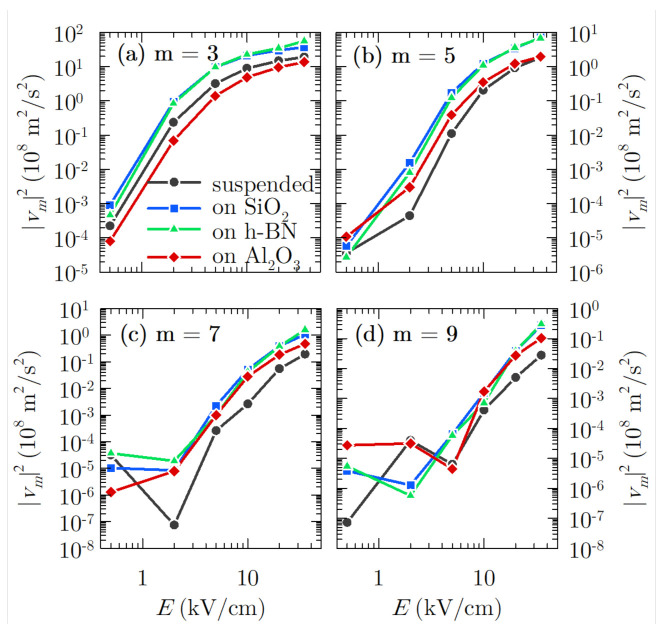
Intensity of the (**a**) third, (**b**) fifth, (**c**) seventh and (**d**) ninth harmonics of the excitation frequency f=300 GHz as a function of the electric field for the four cases under study.

Once the velocity response of the system has been described, we are in better conditions for evaluating the harmonic generation. In Figure 4, the intensity of different harmonics as a function of the electric field amplitude $E_{AC}$ has been represented for the cases under analysis and for an excitation frequency equal to 300 GHz. Only odd harmonics are depicted, since the velocity is an odd periodic function. Harmonic generation starts being appreciable for fields above 2 kV/cm with intensities of the third harmonic between $10^{6}$ and $10^{7}$ m${}^{2}$/s${}^{2}$ at this electric field amplitude, and between $10^{2}$ and $10^{3}$ m${}^{2}$/s${}^{2}$ for the ninth harmonic at 2 kV/cm for the different samples under study. The intensity of the harmonics is lower at this regime than at higher fields due to the sinusoidal-like shape of the velocity response (as we observed in Figure 3c for E${}_{AC}=0.5$ kV/cm). Furthermore, the carriers response is quite linear, consequent with the behaviour observed in the velocity-electric field curves, where saturation starts at about 1 kV/cm (see Figure 2). Therefore, the best conditions for harmonic generation are fostered for electric field amplitudes corresponding to electric fields in the velocity saturation regime in stationary conditions.

Regarding the influence of the substrate, graphene on Al${}_{2}$O${}_{3}$ presents the weakest third harmonic intensity, while for the upper odd harmonics, it is suspended graphene—the one with the lower intensities. At the higher end of the scale, we find that graphene on SiO${}_{2}$ and especially graphene on h-BN reveal the largest harmonic intensity in all of the field range. The strong relaxation effect of the Al${}_{2}$O${}_{3}$ SPPs is related to the weak intensity of the generated harmonics in comparison to the other substrates analysed.

**Figure 5:**
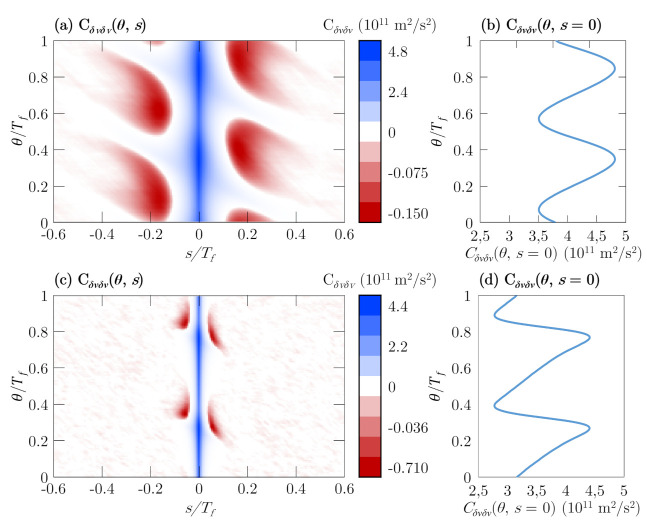
The transient correlation functions for graphene on h-BN${}_{2}$ at (**a**) $E_{AC}=2$ and (**c**) 20 kV/cm. The corresponding correlations at s=0 are also depicted at (**b**,**d**).

In general, an arbitrary number of odd harmonics would be generated. However, as seen in Figure 4, the magnitude decreases with the harmonic order, e.g., around two orders of magnitude between the third and ninth harmonics. Therefore, the feasibility of the harmonic signal extraction procedure will not be complete unless the noise level is taken into account, as the power generated from carrier velocity fluctuations can bury the generated harmonic signal. The first step to evaluate the noise level is to introduce the correlation function of velocity fluctuations $C_{\delta v\delta v}\left(\theta ,s\right)$, which in the case of the present work, is not a single-time function due to the presence of an oscillating excitation electric field, and this becomes a non-stationary process [11,29]. Therefore, the correlation function depends on two times: θ (defined as any instant within the period $T_{f}$ of the signal relative to the excitation frequency) and *s* (known as the correlation time). In Figure 5, the transient correlation function for graphene on a substrate (h-BN is chosen once again due to its better performance) at low and high applied electric field is presented (Figure 5a,c, respectively). As it can be observed, the period of the correlation function regarding θ is equal to half the period of the electric field for symmetry reasons. On the other hand, the function is asymmetric considering *s*; therefore, when comparing the processes (i.e., cooling or heating) that carriers undergo at (s−θ) and (s+θ), we observe that they are different. At s=0, the value of this function corresponds to the variance of velocity fluctuations 〈δv(θ)δv(θ)〉 [11]. This quantity reflects how wide the fluctuations are at that given time inside the signal period, which will be linked to the white noise level, as we will see later.

The evolution of the values of the variance with θ are also depicted in Figure 5b,d for the same electric fields for a better understanding. The maxima of the variance corresponds to the zero-crossing of the drift velocity response. On the other hand, the variance minima are linked to the instants when 〈v(θ)〉 presents the drift velocity peaks (in absolute value). This fact differs from III–V materials, where the maximum approximately corresponds to the moment when the field reaches its maximum value, and the minima appear almost when the electric field equals 0 [11]. At $E_{AC}=20$ kV/cm (see Figure 5d), we observe that although the amplitude of the fluctuation is larger, the values of the correlation function reached are smaller; this will ultimately have an influence over the spectral density at low-frequency, presenting a lower value as $E_{AC}$ rises, as we will observe afterwards.

Concerning the effect of increasing the electric field amplitude over the correlation function, its maximum value decreases and the minimum is shallower (see Figure 5c). Moreover, the decay from the maxima to the minima occurs in shorter correlation times. This behaviour is explained by the increase in the average carrier energy and the number of scattering mechanisms as the electric field rises, acting therefore as a correlation-breaker. Attending to the different cases under study, the correlation function for graphene on SiO${}_{2}$ presents a similar behaviour than on h-BN, while on Al${}_{2}$O${}_{3}$ at high field conditions, where the greatest amount of SPP is suffered, the drop from the maxima to the minima is larger. On the other hand, the correlation is lost earlier in the suspended case, where the importance of the carrier–carrier interaction is the most important. The fastest or softest drop of the correlation function will have a direct consequence over the spectral density, which is an important parameter for understanding the noise level, as we will se below.

The instantaneous power spectral density of velocity fluctuations is determined from the Fourier transform of the correlation function with respect to *s*. Furthermore, the mean spectral density is the result of averaging this latter quantity over all θ along the AC field period:

(4) $${\bar{S}}_{\delta v\delta v}\left(\nu \right)=1/T_{f}\int_{0}^{T_{f}}\int_{-\infty }^{\infty }C_{\delta v\delta v}\left(\theta ,s\right)\exp\left(2\pi i\nu s\right)dsd\theta$$

**Figure 6:**
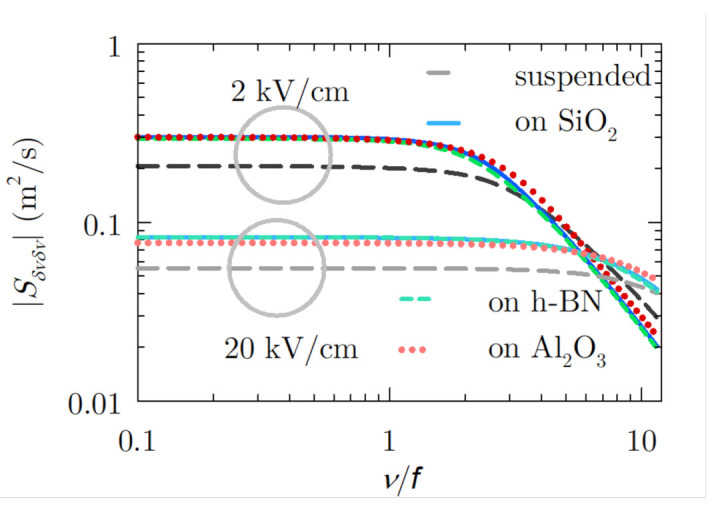
Noise spectrum of velocity fluctuations as a function of the frequency normalised to that of the AC field for all the considered cases with E${}_{AC}=2$ and 20 kV/cm.

This quantity, related to the dissipated power induced by velocity fluctuations at a given frequency, is depicted in Figure 6 as a function of the frequency normalised to that of the AC field (300 GHz) and two values of electric field amplitude. The normalisation of the frequency will allow us to keep the reference of the position of the generated harmonics that we will analyse next. It presents a Lorentzian shape for all the cases under study at the whole applied electric field range. III–V semiconductors also present this characteristic shape at low electric fields, but for higher fields, a peak appears related to the intervalley transfer [11], which does not apply to the graphene case. We observe that this characteristic white noise plateau extends up to larger frequency values when the electric field amplitude increases, while the value of ${\bar{S}}_{\delta v\delta v}\left(\nu \right)$ at low-frequency values diminishes as a consequence of the shorter relaxation times of velocity fluctuations. After the corner, a monotonic decay of the power spectral density starts for all the cases. We also observe that the value of the spectral density for graphene over any of the substrates analysed is larger than in the suspended sample, independently of the electric field amplitude, $E_{AC}$, considered. Beyond the absence of SPP scattering mechanisms in suspended graphene, it is remarkable that the carrier–carrier interaction is the largest, yielding the fastest drop of the correlation and, therefore, a smaller spectral density. Al${}_{2}$O${}_{3}$ is the substrate that causes graphene to have the lower scattering activity of this type; however, the large presence of SPP scattering mechanisms counterbalances this, and the noise level ends up being similar to that obtained with the other two substrates.

On the other hand, we can affirm for the examined samples that harmonic detection may be more complex at low fields since the white noise level at low frequencies is larger, as we can observe in Figure 6. This behaviour of the white noise level has also been evidenced for suspended graphene under a static applied field [24]. However, it is the opposite in comparison to III–V semiconductors, where the white noise level increases with the applied field [11]. This can be considered as an advantage of graphene over III–V semiconductors at high electric fields. Regarding the effect of increasing the frequency, at about ν=6f, similar noise levels appear independently of the field. Meanwhile, at elevated frequencies, the noise level is larger for higher field values, and this strong existing noise competes with efficient generation over the seventh harmonic, as we shall see now.

**Figure 7:**
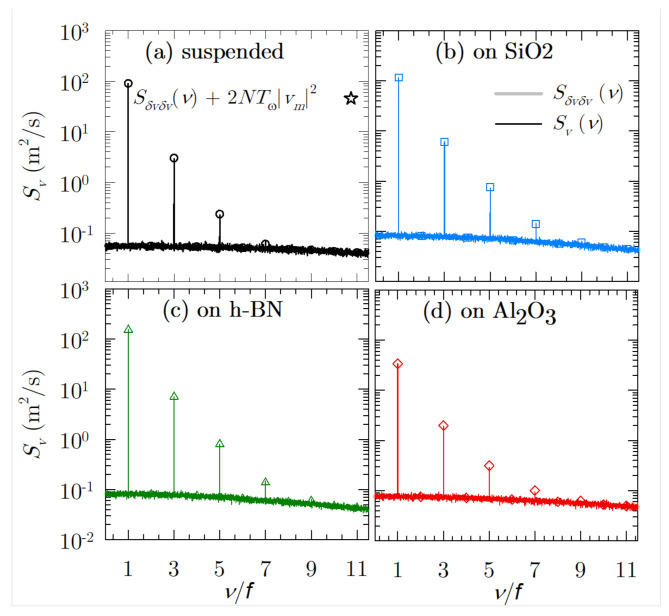
Full spectrum of velocity response for all the considered cases with E${}_{AC}=20$ kV/cm and N = 300 cycles.

A comparison between the noise level and the generated harmonics is reported in Figure 7, where the spectrum of the velocity response is depicted. This study has been developed at f=300GHz for the different cases under consideration and high electric field amplitude conditions ($E_{AC}=20$ kV/cm), since at low fields almost all the harmonics are screened by the intrinsic noise, except the one corresponding to the fundamental frequency. The exposure time of a sensor to the radiation under consideration was set as an integer multiple of the fundamental period, $T=NT_{f}$, with N=300. This is relative to a minimum bandwidth of 1 GHz. We are able to obtain the velocity response in two different ways: correlation function combined with Fourier coefficients, and the discrete Fourier transform [5], both showing a very good fitting.

As it can be seen, the spectral density due to velocity fluctuations presents a flat shape, as already seen in [5] for a large static electric field. Moreover, the seventh harmonic is still discernible for the cases of graphene on h-BN and SiO${}_{2}$, while only up to the fifth harmonic can the suspended sample and the one on Al${}_{2}$O${}_{3}$ be distinguished. The number of harmonics generated for graphene on h-BN and SiO${}_{2}$ is similar to those obtained for III–V materials, such as InP [11]. However, we must keep in mind that in the discussion presented in [11], the conditions are different: lower electric field amplitude (with $E_{AC}=8\;\mathrm{kV}/\mathrm{cm}$, which yields lower noise in III–V materials as previously stated), lower excitation frequency (f=200GHz) lower temperature (at 80 K), and finally, hot phonon effects and carrier–carrier interactions were not included in [11].

From this comparison, we are able to assert that graphene on substrates, such as SiO${}_{2}$ and mainly h-BN, may present better potential for high-order harmonic generation than III–V materials when considering the high amplitude of electric field and larger exposure times. The better performance of graphene on h-BN is directly related to the larger values of phonon energies considered for this substrate as compared to the other ones (see Table 1); in addition, from the analysis of the SPP scattering probability as a function of the energy (as shown in [26] for initial photoexcited conditions), we observe that the limit of appearance of the SPP emission for h-BN arises at larger carrier energies than for the rest of them, thus meaning that the carriers can accelerate faster.

At this point, it is interesting to determine the threshold bandwidth for the cases under study, defined as the bandwidth below which it is not possible to extract the *m*th harmonic from the background noise [11]. This implies that the bandwidth resolution of a detector must be lower than this quantity in order to discern the harmonics from the existing noise level:

(5) $$\Delta \nu_{th}=\frac{2|v_{m}{|}^{2}}{{\bar{S}}_{\delta v\delta v}\left(\nu_{m}\right)}$$

**Figure 8:**
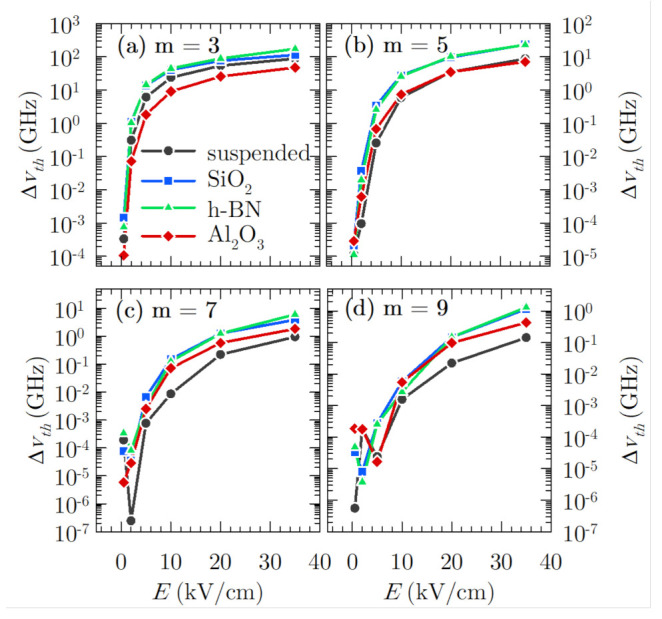
Threshold bandwidth for the (**a**) third, (**b**) fifth, (**c**) seventh, and (**d**) ninth harmonics of the excitation frequency f=300 GHz in the four cases under study as a function of the electric field and a temperature of 300 K.

This function is shown in Figure 8 for the third to the ninth harmonics for all the samples considered in the present work as a function of the applied electric field. As it can be observed, for fields lower than 5 kV/cm, the threshold bandwidths are similar for the different cases considered. For larger electric fields, graphene on h-BN generally presents a wider bandwidth, being equal to 174, 23, 6 and 1.3 GHz for each odd harmonic from third to ninth order, respectively, under the largest electric field amplitude analysed in the present work (35 kV/cm). It is closely followed by the results obtained by graphene on SiO${}_{2}$, while Al${}_{2}$O${}_{3}$ presents the smallest threshold bandwidth at m=3, surpassing the values obtained by suspended graphene for the upper harmonics. These results are coherent with the previous discussion for the intensity of the harmonics and the behaviour of the power spectral density, since h-BN and also SiO${}_{2}$ present the most relevant intensity of the harmonics, and Al${}_{2}$O${}_{3}$ the lowest at m=3. For upper harmonics, suspended graphene demands a narrower bandwidth for harmonic detection over the noise level. On the other hand, these threshold bandwidths are similar to those required in III–V semiconductors as shown in [11], although it must be taken into account that in this latter case, the fundamental frequency of the applied electric field and the temperature are lower (200 GHz and 80 K), both facts favouring a better performance. Furthermore, the elision in that work of hot phonon effects and carrier–carrier interactions may also boost those results, since they are a great source of velocity fluctuations and, hence, of background noise.

## 4. Conclusions

To summarise, we have used our Monte Carlo simulator to elucidate which substrate provides better performance for high order harmonic generation in graphene. The results suggest that graphene on h-BN not only presents a better velocity response but is also the best candidate for high order harmonic extraction as long as strong enough fields are applied, while Al${}_{2}$O${}_{3}$ substrate is generally the worst case. It has also been observed that the performance of graphene on the different substrates is potentially superior to that of III–V semiconductors at room temperature, highlighting that, contrarily to these materials, at high electric field amplitude, not only is harmonic generation promoted, but there is also is a reduction of the noise level, evidenced by the decrease in the correlation function and, consequently, of the spectral density.

## Figures and Tables

**Table 1:** Phonon energies and low and high-frequency dielectric constants considered for the different substrates studied in the present work. The values have been taken from [22,23].

Dielectric	$\epsilon_{0}$	$\epsilon_{\infty }$	$\hbar \omega_{{\mathrm{SPP}}_{1}}$ (meV)	$\hbar \omega_{{\mathrm{SPP}}_{2}}$ (meV)
h-BN	5.09	4.1	101.7	195.7
SiO${}_{2}$	3.9	2.5	59.98	146.51
Al${}_{2}$O${}_{3}$	12.53	3.2	55.01	94.29

**Table 2:** Number of the most relevant scattering mechanisms per unit time, i.e., carrier–carrier (e–e) interaction and SPP, suffered by the carriers on the different samples under study at $E_{AC}=0.5$ kV/cm. In addition, the total scattering events are reported.

Substrate	e–e (s${}^{-1}$)	SPP (s${}^{-1}$)	Total (s${}^{-1}$)
h-BN	$1.60\cdot 10^{12}$	$2.45\cdot 10^{11}$	$1.99\cdot 10^{12}$
SiO${}_{2}$	$1.67\cdot 10^{12}$	$8.23\cdot 10^{11}$	$2.63\cdot 10^{12}$
Al${}_{2}$O${}_{3}$	$1.22\cdot 10^{12}$	$8.16\cdot 10^{12}$	$9.51\cdot 10^{12}$
Suspended	$2.43\cdot 10^{12}$	-	$2.64\cdot 10^{12}$

**Table 3:** Number of the most relevant scattering mechanisms per unit time, i.e., carrier–carrier interaction and SPP, suffered by the carriers on the different samples under study at $E_{AC}=20$ kV/cm. Furthermore, the total scattering events are reported.

Substrate	e–e (s${}^{-1}$)	SPP (s${}^{-1}$)	Total (s${}^{-1}$)
h-BN	$8.34\cdot 10^{12}$	$8.89\cdot 10^{12}$	$2.13\cdot 10^{13}$
SiO${}_{2}$	$8.48\cdot 10^{12}$	$1.39\cdot 10^{13}$	$2.56\cdot 10^{13}$
Al${}_{2}$O${}_{3}$	$3.55\cdot 10^{12}$	$4.32\cdot 10^{13}$	$4.77\cdot 10^{13}$
Suspended	$1.66\cdot 10^{13}$	-	$2.50\cdot 10^{13}$

## Data Availability

The data that support the findings of this study and further information are available on request from the corresponding author.
